# Supplementary material for: Learning induced neuronal identity switch in the superficial layers of the primary somatosensory cortex
Source: bioRxiv. 2023 Sep 1:2023.08.30.555603. Preprint. [Version 1] doi: 10.1101/2023.08.30.555603 (PMC10491147; doi:10.1101/2023.08.30.555603)
Supplement: Supplement 1 [file NIHPP2023.08.30.555603v1-supplement-1.pdf]

bioRxiv preprint doi: https://doi.org/10.1101/2023.08.30.555603; this version posted September 1, 2023. The copyright holder for this preprint (which was not certified by peer review) is the author/funder, who has granted bioRxiv a license to display the preprint in perpetuity. It is made available under aCC-BY 4.0 International license.

Fig. s1

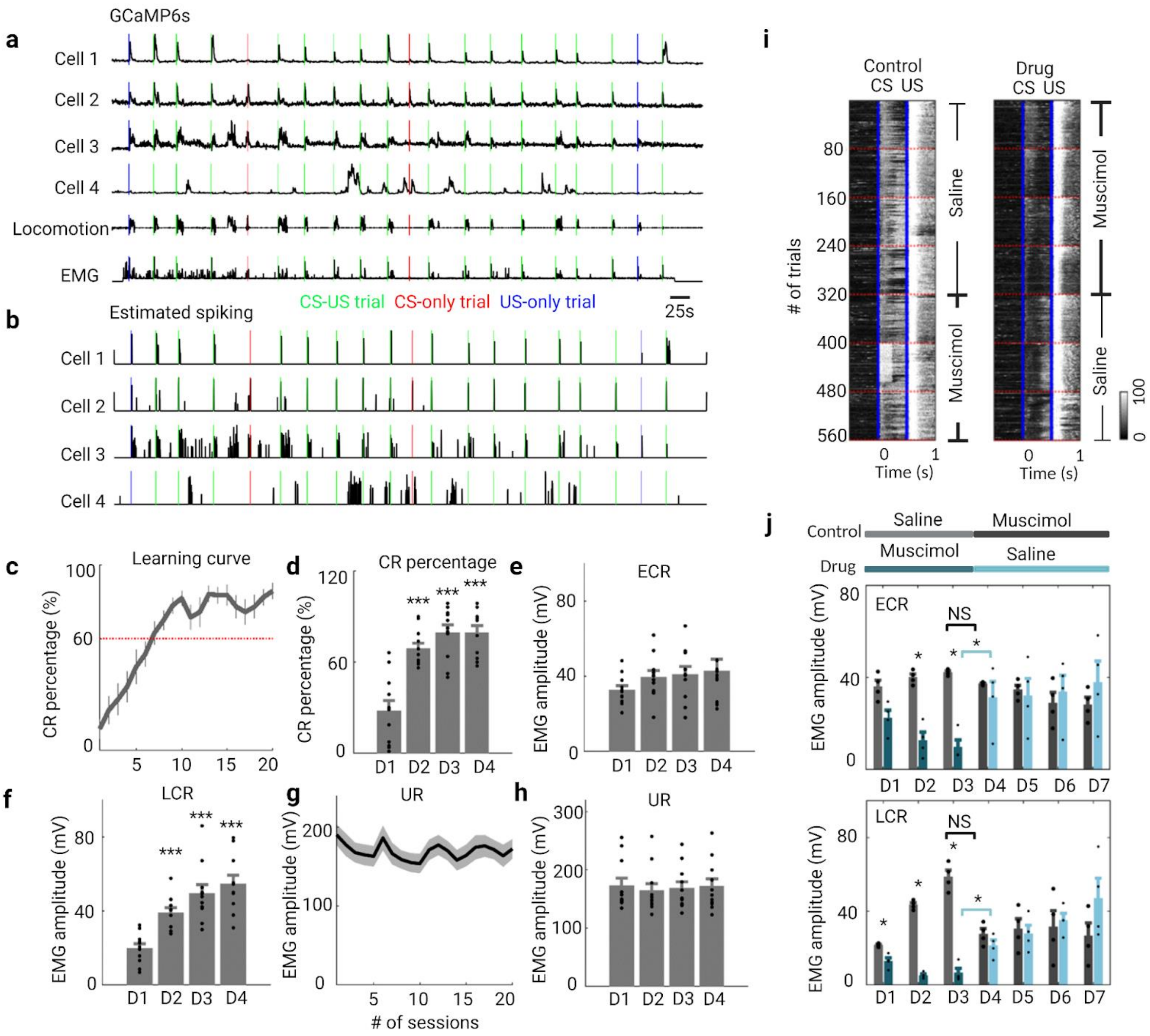

Fig. S1 (Related to Fig. 1): Additional analyses of neural population responses and behavioral patterns

a, GCaMP6s signal (Δf/f) traces from four example L2/3 neurons, locomotion and EMG traces in the first session of TEC training. **b**, Estimated spiking of the example neurons in the first session of TEC training (green, CS-US; red, CS-only; blue, US-only). **c**, Evolution of conditional response percentage (CR%, top) and UR amplitude (bottom) across 20 sessions. Data are shown as mean ± s.e.m. Shaded areas represent s.e.m. **d~h**, Behavior performance during training: CR percentage (n=11 mice; D1 vs. D2, ***P=9.1 × 10^{-4}; D1 vs. D3, ***P=3.0 × 10^{-4} ; D1 vs. D4, ***P=3.0 × 10^{-4}, Wilcoxon test), ECR (n=11 mice; D1 vs. D2, P=0.60; D1 vs. D3, P=0.84; D1 vs. D4, P=0.95, Wilcoxon test), LCR (n=11 mice; D1 vs. D2, ***P=5.0 × 10^{-4}; D1 vs. D3, ***P=1.4 × 10^{-4} ; D1 vs. D4, ***P=1.1 × 10^{-4}, Wilcoxon test) and UR (n=11 mice; D1 vs. D2, P=0.60; D1 vs. D3, P=0.84; D1 vs. D4, P=0.95, Wilcoxon test). Data are shown as mean ± s.e.m. Shaded areas represent s.e.m. **i**, Heat map of EMG responses from representative mice in control (left) and drug (right) groups during TEC learning (16 CS-US trials in one session, five sessions in a day shown in red dashed line; blue solid lines indicate onset of CS or US). The right side of each heat map indicates the treatment of muscimol or saline. **j**, Evolution of EMG amplitude (ECR, top; LCR, bottom) across 7 days (35 sessions) of training in the control group (saline was delivered during the first 3 days and then muscimol was delivered) and drug group (muscimol was delivered during the first 3 days and then saline was delivered) (n=4 in each group; ECR, control vs.

bioRxiv preprint doi: https://doi.org/10.1101/2023.08.30.555603; this version posted September 1, 2023. The copyright holder for this preprint (which was not certified by peer review) is the author/funder, who has granted bioRxiv a license to display the preprint in perpetuity. It is made available under aCC-BY 4.0 International license.

drug in D1, P=0.057; control vs. drug in D2, *P=0.029; control vs. drug in D3, *P=0.029; control vs. drug in D4, P=0.89; control vs. drug in D5, P=0.89; control vs. drug in D6, P=0.49; control vs. drug in D7, P=0.49; control in D3 vs. control in D4, P=0.058; drug in D3 vs. drug in D4, P=0.029; LCR, control vs. drug in D1, *P=0.029; control vs. drug in D2, *P=0.029; control vs. drug in D3, *P=0.029; control vs. drug in D4, P=0.20; control vs. drug in D5, P=0.89; control vs. drug in D6, P=0.89; control vs. drug in D7, P=0.34; control in D3 vs. control in D4, P=0.057; drug in D3 vs. drug in D4, P=0.029; Wilcoxon test).

bioRxiv preprint doi: https://doi.org/10.1101/2023.08.30.555603; this version posted September 1, 2023. The copyright holder for this preprint (which was not certified by peer review) is the author/funder, who has granted bioRxiv a license to display the preprint in perpetuity. It is made available under aCC-BY 4.0 International license.

Fig. s2

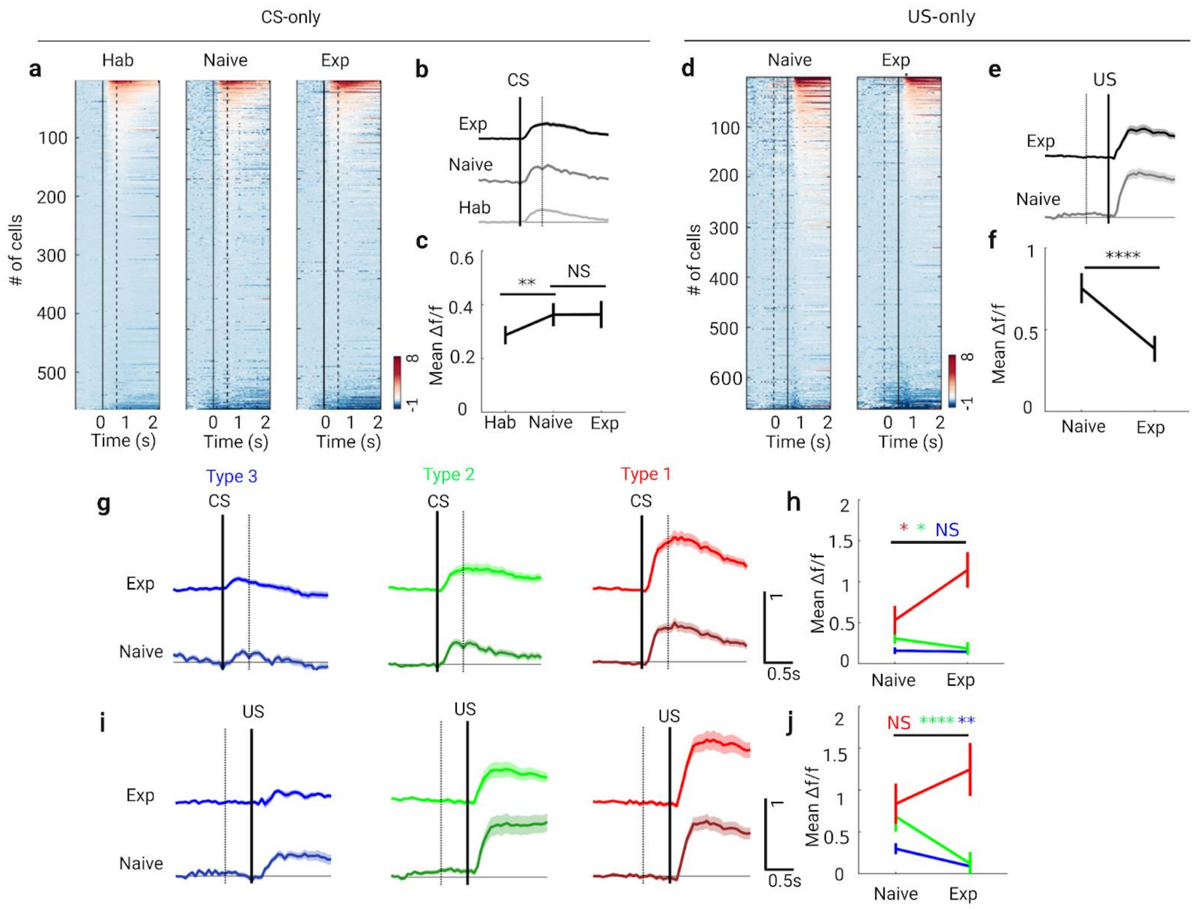

Fig. S2 (Related to Fig. 1): Additional analyses of neural population responses and three types of PNs in CS-only and US-only trials

a, Heat maps of GCaMP6s signals (Δf/f) for PNs in CS-only trials at different stages of training (hab, n=566 neurons from 9 mice; Naive, n=663 neurons from 9 mice; Exp, n=665 neurons from 9 mice). Signals are aligned to the CS onset. **b**, Mean signal traces of PNs in CS-only trials at each learning stage. Data are shown as mean ± s.e.m. Shaded areas represent s.e.m. **c**, CR activity of PNs in CS-only trials at different training stages (hab vs. naive,**P=0.0061, naive vs. exp, NS P=0.99; Wilcoxon test). **d**, Heat maps of GCaMP6s signals (Δf/f) for PNs in US-only trials at different stages of training (Naive, n=663 neurons from 9 mice; Exp, n=665 neurons from 9 mice). Signals are aligned to the CS onset. **e**, Mean signal traces of PNs in US-only trials at each learning stage. Data are shown as mean ± s.e.m. Shaded areas represent s.e.m. **f**, UR activity of PNs in US-only trials at different training stages (naive vs. exp, ****P=3.62 × 10^{-14}; Wilcoxon test). **g**, Mean signal traces of three subtypes of PNs in CS-only trials at each learning stage. Data are shown as mean ± s.e.m. Shaded areas represent s.e.m. **h**, CR activity of three subtypes of PNs in CS-only trials at different training stages (naive vs. exp in type 1,*P=0.011, naive vs. exp in type 2,*P=0.049, naive vs. exp in type 3, NS P=0.95; Wilcoxon test). **i**, Mean signal traces of three subtypes of PNs in US-only trials at each learning stage. Data are shown as mean ± s.e.m. Shaded areas represent s.e.m. **j**, UR activity of three subtypes of PNs in US-only trials at different training stages (naive vs. exp in type 1, NS P=0.84, naive vs. exp in type 2,**P=0.0014, naive vs. exp in type 3, ****P=3.04 × 10^{-9}; Wilcoxon test).

bioRxiv preprint doi: https://doi.org/10.1101/2023.08.30.555603; this version posted September 1, 2023. The copyright holder for this preprint (which was not certified by peer review) is the author/funder, who has granted bioRxiv a license to display the preprint in perpetuity. It is made available under aCC-BY 4.0 International license.

Fig. s3

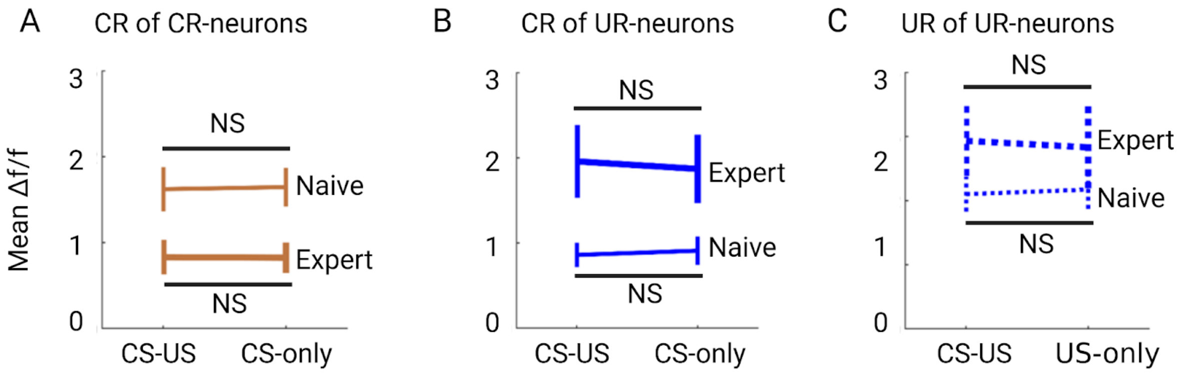

Fig. S3 (Related to Fig. 2): Additional analyses of CR-neurons and UR-neurons in CS-only and US-only trials

a, Comparison of CR-neurons' CS-induced responses (CR) in CS-US trials and CS-only trials at naive and expert stages (naive, NS P=0.88; expert, NS P=0.41; Wilcoxon test). **b**, Comparison of UR-neurons' CS-induced responses (CR) in CS-US trials and CS-only trials at naive and expert stages (naive, NS P=0.36; expert, NS P=0.91; Wilcoxon test). **c**, Comparison of UR-neurons' US-induced responses (UR) in CS-US trials and CS-only trials at naive and expert stages (naive, NS P=0.68; expert, NS P=0.18; Wilcoxon test). Data are shown as mean ± s.e.m.

bioRxiv preprint doi: https://doi.org/10.1101/2023.08.30.555603; this version posted September 1, 2023. The copyright holder for this preprint (which was not certified by peer review) is the author/funder, who has granted bioRxiv a license to display the preprint in perpetuity. It is made available under aCC-BY 4.0 International license.

Fig. s4

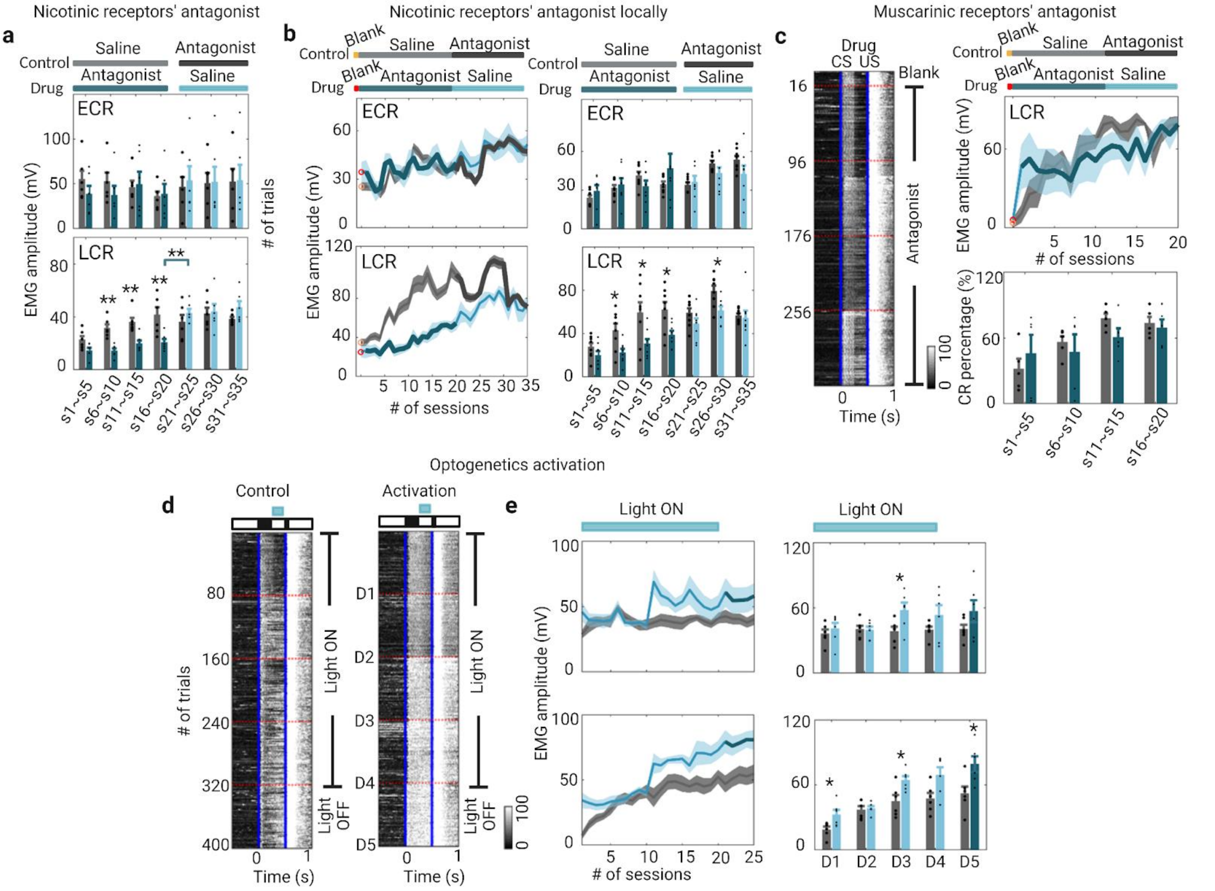

Fig. S4 (Related to Fig. 3): EMG changes after pharmacological and optogenetic manipulation

a, EMG amplitudes (ECR, top; LCR, bottom) of nAChR antagonist and control groups (n=6 mice in each group; ECR, drug vs. control in s1~s5, P=0.31, drug vs. control in s6~s10, P=0.13, drug vs. control in s11~s15, P=1, drug vs. control in s16~s20, P=0.82, drug vs. control in s21~s25, P=0.94, drug vs. control in s26~s30, P=0.94, drug vs. control in s31~s35, P=1; LCR, control in s1~s5, P=0.093, drug vs. control in s6~s10, **P=0.0022, drug vs. control in s11~s15, **P=0.0087, drug vs. control in s16~s20, **P=0.0087, drug vs. control in s21~s25, P=0.31, drug vs. control in s26~s30, P=0.82, drug vs. control in s31~s35, P=0.13; Wilcoxon test). **b**, EMG changes after local treatment of nAChR antagonist and saline in S1. Left, evolution of EMG amplitude (ECR, top; LCR, bottom) across 35 sessions of training in the control group (One blank session in day 0, saline was delivered during the first 3 days and then nAChR antagonist was delivered) and drug group (One blank session in day 0, nAChR antagonist was delivered during the first 3 days and then saline was delivered) (n=8 in each group). Right, EMG amplitudes (ECR, top; LCR, bottom) of nAChRs antagonist and control groups (n=8 mice in each group; ECR, drug vs. control in s1~s5, P=0.16, drug vs. control in s6~s10, P=0.72, drug vs. control in s11~s15, P=0.23, drug vs. control in s16~s20, P=0.51, drug vs. control in s21~s25, P=0.72, drug vs. control in s26~s30, P=0.44, drug vs. control in s31~s35, P=0.16; LCR, control in s1~s5, P=0.19, drug vs. control in s6~s10,

bioRxiv preprint doi: https://doi.org/10.1101/2023.08.30.555603; this version posted September 1, 2023. The copyright holder for this preprint (which was not certified by peer review) is the author/funder, who has granted bioRxiv a license to display the preprint in perpetuity. It is made available under aCC-BY 4.0 International license.

*P=0.038, drug vs. control in s11~s15, *P=0.049, drug vs. control in s16~s20, *P=0.021, drug vs. control in s21~s25, P=0.19, drug vs. control in s26~s30, *P=0.038, drug vs. control in s31~s35, P=0.23; Wilcoxon test).Data are shown as mean ± s.e.m. Shaded areas represent s.e.m. **c**, Left, heat map of EMG responses from muscarinic receptor (mAChR) antagonist treated mouse during TEC learning (16 CS-US trials in one session, five sessions in a day shown in red dashed line; blue solid lines indicate onset of CS or US). The right side of the heat map indicates the treatment of mAChR antagonist. Data are shown as mean ± s.e.m. Shaded areas represent s.e.m. Right, LCR (top) and CR percentage (bottom) changes of mAChRs antagonist and control group (n=5 mice in each group; ECR, drug vs. control in s1~s5, P=0.69, drug vs. control in s6~s10, P=0.69, drug vs. control in s11~s15, P=0.10, drug vs. control in s16~s20, P=0.75). **d**, EMG changes after optogenetic activation of cholinergic fibers in S1 (16 CS-US trials in one session, five sessions in a day shown in red dashed line; blue solid lines indicate onset of CS or US). The right side of the heat map indicates light ON and light OFF. **e**, Left, evolution of EMG amplitude (ECR, top; LCR, bottom) across 25 sessions of training in the control group (light ON during the first 3 days and then light OFF) and activation group (light ON during the first 3 days and then light OFF) (n=6 in each group). Right, EMG amplitudes (ECR, top; LCR, bottom) of activation and control groups (n=6 mice in each group; ECR, activation vs. control in D1, P=0.31, activation vs. control in D2, P=0.94, activation vs. control in D3, *P=0.041, activation vs. control in D4, P=0.31, activation vs. control in D5, P=0.39; LCR, activation vs. control in D1, *P=0.041, activation vs. control in D2, P=0.94, activation vs. control in D3, *P=0.015, activation vs. control in D4, P=0.065, activation vs. control in D5, *P=0.015; Wilcoxon test). Data are shown as mean ± s.e.m. Shaded areas represent s.e.m.

bioRxiv preprint doi: https://doi.org/10.1101/2023.08.30.555603; this version posted September 1, 2023. The copyright holder for this preprint (which was not certified by peer review) is the author/funder, who has granted bioRxiv a license to display the preprint in perpetuity. It is made available under aCC-BY 4.0 International license.

Fig. s5

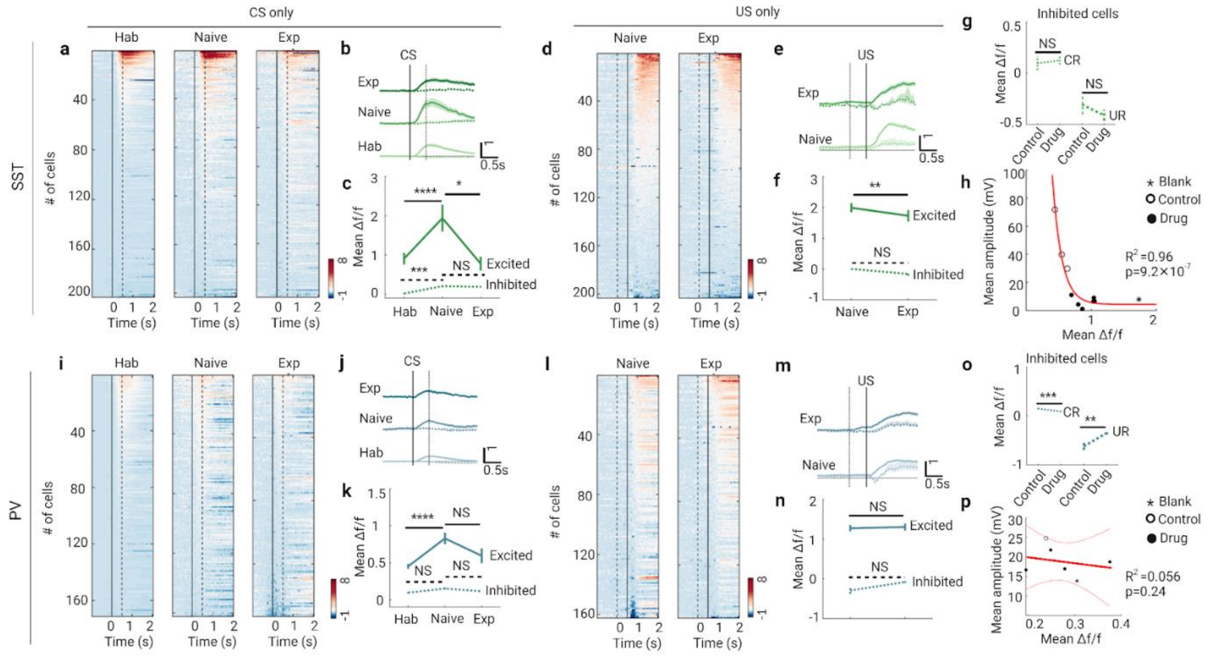

Fig. S5 (Related to Fig. 4): Additional analyses of SST- and PV- INs.

a, Heat maps of GCaMP6s signals (Δf/f) for SST-INs in CS-only trials at different stages of training (Hab, n=198 neurons from 7 mice; Naive, n=203 neurons from 7 mice; Exp, n=255 neurons from 7 mice). Signals are aligned to the CS onset. **b**, Mean signal traces of SST-INs in CS-only trials at each learning stage (solid line indicates the excited cells, dashed line indicates the inhibited cells). Data are shown as mean ± s.e.m. Shaded areas represent s.e.m. **c**, CR activity of excited (solid line) and inhibited (dashed line) SST-INs in CS-only trials at different training stages (CR of excited cells, hab vs. naive, ****$P=1.97 \times 10^{-7}$, naive vs. exp, *P=0.027; CR of inhibited cells, hab vs. naive, ***$P=3.63 \times 10^{-4}$, naive vs. exp, NS P=0.90; Wilcoxon test). **d**, Heat maps of GCaMP6s signals (Δf/f) for SST-INs in US-only trials at different stages of training (Naive, n=203 neurons from 7 mice; Exp, n=255 neurons from 7 mice). Signals are aligned to the CS onset. **e**, Mean signal traces of SST-INs in US-only trials at each learning stage (solid line indicates the excited cells, dashed line indicates the inhibited cells). Data are shown as mean ± s.e.m. Shaded areas represent s.e.m. **f**, UR activity of excited (solid line) and inhibited (dashed line) SST-INs in US-only trials at different training stages (UR of excited cells, **P=0.0050; UR of inhibited cells, NS P=0.053; Wilcoxon test). **g**, CR (fine line) and UR (bold line) activity of inhibited SST-INs in drug and control sessions (CR, NS P=0.44; UR, NS P=0.29; Wilcoxon test). **h**, Mean SST-INs' signal versus mean EMG amplitude from CS onset to US onset in blank (asterisk), drug (solid circle) and control (hollow circle) sessions from a represented mouse (decay function fitting, n= 10 sessions from 1 mice). **i**, Heat maps of GCaMP6s signals (Δf/f) for PV-INs in CS-only trials at different stages of training (Hab, n=172 neurons from 5 mice; Naive, n=162 neurons from 5 mice; Exp, n=153 neurons from 5 mice). Signals are aligned to the CS onset. **j**, Mean signal traces of PV-INs in CS-only trials at each learning stage (solid line indicates the excited cells, dashed line indicates the inhibited cells). Data are shown as mean ± s.e.m. Shaded areas represent s.e.m. **k**, CR activity of excited (solid line) and inhibited (dashed

bioRxiv preprint doi: https://doi.org/10.1101/2023.08.30.555603; this version posted September 1, 2023. The copyright holder for this preprint (which was not certified by peer review) is the author/funder, who has granted bioRxiv a license to display the preprint in perpetuity. It is made available under aCC-BY 4.0 International license.

line) PV-INs in CS-only trials at different training stages (CR of excited cells, hab vs. naive, ****P=6.72 × 10^{-6}, naive vs. exp, P=0.099; CR of inhibited cells, hab vs. naive, NS P=0.11, naive vs. exp, NS P=0.67; Wilcoxon test). **l**, Heat maps of GCaMP6s signals (Δf/f) for PV-INs in US-only trials at different stages of training (Naive, n=162 neurons from 5 mice; Exp, n=153 neurons from 5 mice). Signals are aligned to the CS onset. **m**, Mean signal traces of PV-INs in US-only trials at each learning stage (solid line indicates the excited cells, dashed line indicates the inhibited cells). Data are shown as mean ± s.e.m. Shaded areas represent s.e.m. **n**, UR activity of excited (solid line) and inhibited (dashed line) PV-INs in US-only trials at different training stages (UR of excited cells, P=0.65; UR of inhibited cells, NS P=0.47; Wilcoxon test). **o**, CR (fine line) and UR (bold line) activity of inhibited PV-INs in drug and control sessions (CR, ***P=7.78 × 10^{-4}; UR, **P=0.0033; Wilcoxon test). **p**, Mean PV-INs signal versus mean EMG amplitude from CS onset to US onset in blank (asterisk), drug (solid circle) and control (hollow circle) sessions from a represented mouse(Pearson correlation fit and 95% confidence bands, n= 6 sessions from 1 mice).
